# Intervention *versus* surveillance in patients with common bile duct stones detected by intraoperative cholangiography: a population-based registry study

**DOI:** 10.1093/bjs/znab324

**Published:** 2021-10-13

**Authors:** E Johansson, J Österberg, E Sverdén, L Enochsson, G Sandblom

**Affiliations:** 1 Department of Clinical Science and Education Södersjukhuset, Karolinska Institutet, Stockholm, Sweden; 2 Centre for Clinical Research, Uppsala University, Falun, Sweden; 3 Department of Urology, Karolinska University Hospital, Stockholm, Sweden; 4 Department of Clinical Sciences, Intervention and Technology (CLINTEC), Karolinska Institutet, Stockholm, Sweden; 5 Department of Surgery, Mora Hospital, Mora, Sweden; 6 Department of Surgery, Södersjukhuset, Stockholm, Sweden; 7 Department of Surgical and Perioperative Sciences, Surgery, Umeå University, Umeå, Sweden

## Abstract

**Background:**

Each year 13 000 patients undergo cholecystectomy in Sweden, and routine intraoperative cholangiography (IOC) is recommended to minimize bile duct injuries. The risk of requiring endoscopic retrograde cholangiopancreatography (ERCP) following cholecystectomy for common bile duct (CBD) stones where IOC is omitted and in patients with CBD stones left *in situ* is not well known.

**Methods:**

Data were retrieved from the population-based Swedish Registry of Gallstone Surgery and ERCP between 1 January 2009 and 10 December 2019. Primary outcome was risk for postoperative ERCP for retained CBD stones.

**Results:**

A total of 134 419 patients that underwent cholecystectomy were included and 2691 (2.0 per cent) subsequently underwent ERCP for retained CBD stones. When adjusting for emergency or planned cholecystectomy, preoperative symptoms suggestive of CBD stones, sex and age, there was an increased risk for ERCP when IOC was not performed (hazard ratio (HR) 1.4, 95 per cent c.i. 1.3 to 1.6). The adjusted risk for ERCP was also increased if CBD stones identified by IOC were managed with surveillance (HR 5.5, 95 per cent c.i. 4.8 to 6.4). Even for asymptomatic small stones (less than 4 mm), the adjusted risk for ERCP was increased in the surveillance group compared with the intervention group (HR 3.5, 95 per cent c.i. 2.4 to 5.1).

**Conclusion:**

IOC plus an intervention to remove CBD stones identified during cholecystectomy was associated with reduced risk for retained stones and unplanned ERCP, even for the smallest asymptomatic CBD stones.

## Teaser

This population-based registry study shows that when common bile duct (CBD) stones, even the smallest, are identified by intraoperative cholangiography (IOC) and managed by surveillance, there is an increased risk for retained stones requiring endoscopic retrograde cholangiopancreatography (ERCP). For asymptomatic stones less than 4 mm diameter, 10.7 per cent in the surveillance group had a retained stone following surgery. These findings imply that even the smallest CBD stones identified by IOC should be managed with active intervention rather than postoperative surveillance, due to the risk of ERCP being required for stones retained in the CBD.

## Introduction

Approximately 13 000 patients undergo cholecystectomy in Sweden each year. The Swedish Agency for Health Technology Assessment and Assessment of Social Services (SBU) recommends routine intraoperative cholangiography (IOC) to minimize the risk for bile duct injury^1^. IOC has been performed in 90 per cent of all cholecystectomies since 2011 and it has been estimated that seven bile duct injuries per year in Sweden are avoided by routine IOC^2^^,^^3^.

IOC may detect concomitant common bile duct (CBD) stones. CBD stones are diagnosed in 8.6 per cent of planned and 21.0 per cent of emergency cholecystectomies^4^. In 2018, SBU concluded that there was a paucity of data on the need for removal of CBD stones detected during cholecystectomy and this has hindered the development of comprehensive guidelines on the management of CBD stones detected by IOC^5^.

It is unclear whether interventions to remove CBD stones intraoperatively reduce the risk for retained and symptomatic CBD stones after surgery. This is most pertinent for CBD stones with a small diameter (less than 4 mm) with no symptoms of biliary obstruction. The risk for retained CBD stones when left *in situ* is generally considered minimal.

This study assessed the fate of CBD stones left *in situ* and the risk of being retained with the need for subsequent endoscopic retrograde cholangiopancreatography (ERCP) for removal. The secondary aim was to assess if an intraoperative or planned postoperative intervention for CBD stones reduces the risk for retained stones.

## Methods

The study was approved by the Swedish Ethics Review Authority (2019-04224) and was conducted in accordance with the Declaration of Helsinki (revision 2013)^6^. The paper is presented in accordance with the STROBE reporting checklist.

This was a retrospective nationwide registry-based cohort study analysing the national Swedish Registry of Gallstone surgery and Endoscopic Retrograde Cholangiopancreatography (GallRiks). GallRiks was started in 2005 and since 2009 it has included approximately 90 per cent of all cholecystectomies and ERCPs performed in Sweden. GallRiks is validated by cross-linking with the national patient registry, where all procedures are registered, by independent assessors. It has consistently been shown to have 97 per cent accuracy when compared with medical records^7^^,^^8^.

**Fig. 1:**
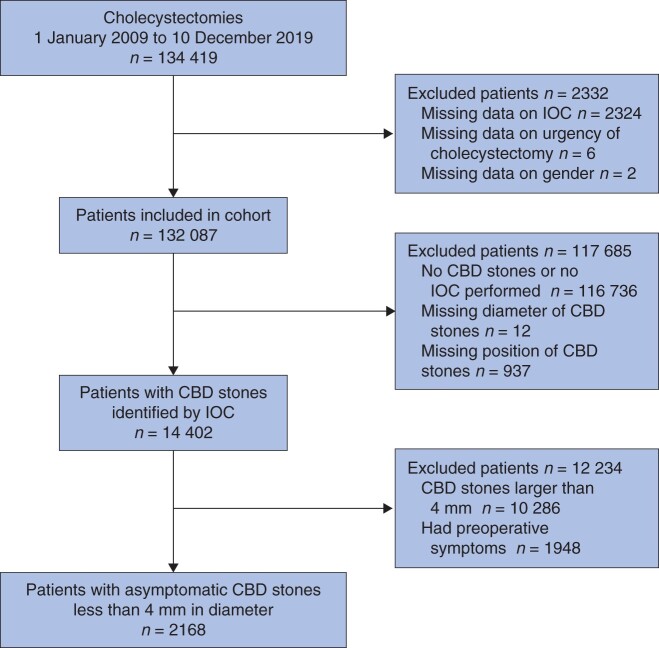
Cohort inclusion diagram IOC, intraoperative cholangiography; CBD, common bile duct.

All index cholecystectomies (laparoscopic, open, and conversion from laparoscopic to open) registered between 1 January 2009 and 10 December 2019 were selected. Patients with missing data for any of the variables in the analyses were excluded as shown in the study flow chart (*Fig. 1*).

The diameter and the location of the gallstones as well as the diameter of the CBD during IOC was estimated by the surgeon and registered in GallRiks. The diameter of the gallstone was categorized as less than 4 mm, 4–8 mm, and more than 8 mm.

The decision to perform an intraoperative or planned postoperative intervention for stone removal was made by the surgeon. The motivation for the decision was not registered in GallRiks. It was presumed that larger stones or stones with signs of biliary obstruction were usually managed by intraoperative removal.

Procedures where IOC was attempted but unsuccessful were included in the ‘IOC not performed’ group because no visualization of the CBD was achieved and thus possible CBD stones remained undetected in this group.

In GallRiks, an intervention for CBD stones was defined as any perioperative measure undertaken to remove a CBD stone, that is, flushing/manipulating with a cholangiography catheter, transcystic extraction, intraoperative or postoperative rendezvous ERCP within 7 days, intraoperative laparoscopic or open choledochotomy. Patients with a guidewire left in the cystic duct and CBD for early postoperative rendezvous ERCP for stone removal (within 7 days following cholecystectomy) were included in the intervention group. The decision to perform a postoperative ERCP was made during cholecystectomy^9–11^.

When CBD stones were identified and ‘no measure taken to remove the CBD stone’ was registered in GallRiks, the patient was categorized as surveillance.

The outcome measure ‘retained CBD stone’, was defined as a CBD stone removed at ERCP performed more than 7 days after cholecystectomy, regardless of the indication for the ERCP.

### Statistical analysis

When estimating the risk for retained stones following procedures where IOC was not undertaken, the entire cohort was included and adjusted for sex, age (above or below median age of 51 years), preoperative symptoms of CBD stone (previous or ongoing jaundice and/or pancreatitis) and emergency or planned cholecystectomy.

The second analysis included patients where IOC was performed, and CBD stones detected. The surveillance group (CBD stones left *in situ*) was compared with the intervention group. Adjustments were made for sex, age, preoperative symptoms of CBD stones, planned or emergency cholecystectomy, CBD stones size (less than 4 mm, 4–8 mm, larger than 8 mm), diameter of the common bile duct (less than 6 mm, 6–10 mm, greater than 10 mm) and location of the CBD stones (common hepatic duct or intrahepatic stones were considered high CBD stones), because size of the stone and location in the bile duct and bile duct diameter could influence the decision to select for intervention or surveillance.

A subgroup analysis was performed for small (less than 4 mm) CBD stones in asymptomatic patients.

Age was categorized as above or below 51 years (median). Common bile duct diameter was categorized as less than 6 mm, 6–10 mm, greater than 10 mm, location of the CBD stone was categorized as low (below cystic duct) or high (above cystic duct).

The cumulative incidence of retained CBD stones for patients that did or did not undergo IOC, as well as patients that underwent a planned intervention or surveillance was analysed using life tables. Cumulative incidence curves were created along with total number and proportion of events in each group.

Potential risk factors for retained CBD stones were analysed with Cox proportional hazards analysis and expressed as hazard ratios (HRs) with 95 per cent confidence intervals. Observation started at the time of the index cholecystectomy, terminal event was the first record of ERCP for retained CBD stones, and death or end of follow-up (10 December 2019) were censored events.

Potential risk factors for retained CBD stones were first analysed with univariable regression and were all included in the multivariable model. No co-variables were excluded in the multivariable model. Unadjusted as well as adjusted estimates were calculated.

Statistical computations were performed using SPSS® (SPSS Statistics for Macintosh, version 27.0. IBM® Corp, Armonk, New York, USA). *P* < 0.050 was considered statistically significant.

## Results

### Patients

Some 134 419 patients underwent cholecystectomy and, after exclusion of patients with missing data, 132 087 patients remained for analyses (*Fig. 1*). The median follow-up time was 5.0 (i.q.r. 2.3 to 7.8 ) years.

In patients with no IOC (16 078 of 132 087 (12.2 per cent), of which 5300 were attempted but unsuccessful) (*Table S1*), fewer patients had preoperative symptoms suggesting CBD stones and less often underwent emergency cholecystectomy. In patients with CBD stones detected by IOC, a larger proportion of patients had preoperative symptoms suggesting CBD stones (jaundice, pancreatitis) and underwent emergency cholecystectomy (*Table 1*).

**Table 1:** Patient characteristics

Characteristic	IOC not performed (*n* = 16 078)	IOC performed, no CBD stones (*n* = 100 658)	IOC performed with CBD stones (*n* = 15 351)	All cholecystectomies (*n* = 132 087)
Age (years)*	52 (19–99)	51 (19–97)	54 (19–97)	51 (19–99)
**Gender**				
Male	6164 (38.3)	33 764 (33.5)	5419 (35.3)	45 347 (34.3)
Female	9914 (61.7)	66 894 (66.5)	9932 (64.7)	86 740 (65.7)
**Preoperative symptoms of CBD stones**†				
No	14 097 (87.7)	82 584 (82.0)	6731 (43.8)	103 412 (78.3)
Yes	1981 (12.3)	18 074 (18.0)	8620 (56)	28 675 (21.7)
**Emergency cholecystectomy**				
No	10 605 (66.0)	69 342 (68.9)	6538 (43)	86 485 (65.5)
Yes	5473 (34.0)	31 316 (31.1)	8813 (56.2)	45 602 (34.5)

Values in parentheses are percentages unless indicated otherwise;

* values are median (range).

† Previous or ongoing jaundice, or pancreatitis. IOC, intraoperative cholangiography; CBD, common bile duct.

The proportion of patients with small (less than 4 mm) CBD stones was larger in the surveillance group than in the intervention group: 63.1 per cent (903 of 1431 patients) *versus* 24.7 per cent (3213 of 12 971 patients) respectively.

For patients with a CBD stone detected during surgery, preoperative symptoms suggestive of CBD stones were recorded in 30.7 per cent (440 of 1431) in the surveillance group and 59.9 per cent (7768 of 12 971) in the intervention group. Emergency cholecystectomy was more common in the intervention group (60.4 per cent, 7839 of 12 971 patients) than the surveillance group (40.0 per cent, 573 of 1431 patients).

There was no difference in CBD stone location between the surveillance and intervention groups (*Tables S2* and *S3*).

### Retained stones

**Fig. 2:**
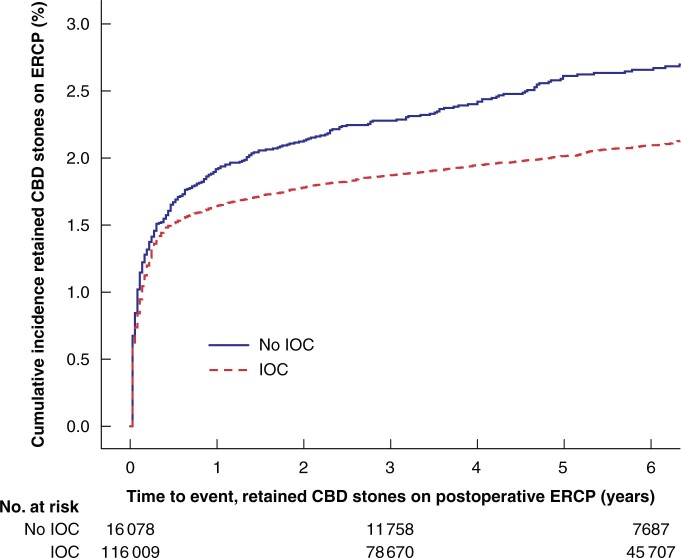
Cumulative incidence of retained common bile duct stones on postoperative endoscopic retrograde cholangiopancreatography Active intervention *versus* surveillance per common bile duct (CBD) stone size. IOC, intraoperative cholangiography; ECRP, endoscopic retrograde cholangiopancreatography.

Some 2691 of 132 087 (2.0 per cent) patients underwent an unplanned ERCP for CBD stones. In patients who underwent cholecystectomy with IOC, 2291 of 116 009 (2.0 per cent) had retained CBD stones compared with 400 of 16 078 (2.5 per cent) in patients who underwent cholecystectomy without IOC (*Table S4*). The HR for retained CBD stones was 1.4 (95 per cent c.i. 1.3 to 1.6) when IOC was not performed, adjusted for preoperative symptoms, emergency cholecystectomy, sex and age. The unadjusted HR was 1.2 (95 per cent c.i. 1.1 to 1.4) (*Table 2* and *Fig. 2*).

**Table 2:** Cox proportional hazards analysis with endoscopic retrograde cholangiopancreatography for retained common bile duct stones (all patients)

	Patients (*n* = 132 087)	Univariable analysis		Multivariable analysis	
		Hazard ratio of retained CBD stones*	*P*	Hazard ratio of retained CBD stones*	***P***
**IOC**					
IOC performed (reference)	116 009 (87.8)	-	-	-	
IOC not performed	16 078 (12.2)	1.2 (1.1, 1.4)	<0.005	1.4 (1.3, 1.6)	<0.005
**Emergency cholecystectomy**					
No (reference)	86 485 (65.5)	-	-	-	-
Yes	45 602 (34.5)	2.4 (2.2, 2.6)	<0.005	1.9 (1.7, 2.0)	<0.005
**Preoperative symptoms of CBD stones**†					
No (reference)	103 412 (78.3)	-	-	-	-
Yes	28 675 (21.7)	3.4 (3.1, 3.6)	<0.005	2.8 (2.6, 3.0)	<0.005
**Gender**					
Female (reference)	45 347 (34.3)	-	-	-	-
Male	86 740 (65.7)	1.4 (1.3, 1.5)	<0.005	1.1 (1.0, 1.2)	0.100
**Age**‡					
Less than cohort median (reference)	66 778 (50.6)	-	-	-	-
Greater than cohort median	65 309 (49.4)	1.8 (1.7, 2.0)	<0.005	1.6 (1.5, 1.8)	<0.005

Values in parentheses are percentages unless indicated otherwise;

* values in parentheses are 95 per cent confidence intervals.

† Previous or ongoing jaundice or pancreatitis.

‡ Median age for the entire cohort was 51 years. IOC, intraoperative cholangiography; CBD, common bile duct.

Of the 14 402 patients who had CBD stones at IOC, 1320 (9.2 per cent) had a retained stone during the follow-up period. In patients that had a perioperative intervention, 1027 of 12 971 patients (7.9 per cent) still had a retained stone *versus* 293 of 1431 (20.5 per cent) in the surveillance group (*Table S5*). Adjusted HR for retained stone was 5.5 (95 per cent c.i. 4.8 to 6.4) when comparing surveillance with intervention. Unadjusted HR was 2.8 (95 per cent c.i. 2.5 to 3.2) (*Table 3*).

**Table 3:** Cox proportional hazards analysis with endoscopic retrograde cholangiopancreatography for retained common bile duct stones

	Patients with CBD stones identified by IOC (*n* = 14 402)	Univariable analysis		Multivariable analysis	
		Hazard ratio of retained CBD stones*	*P*	Hazard ratio of retained CBD stones*	*P*
**CBD stones management**					
Intervention (reference)	12 971 (90.1)	-	-	-	-
Surveillance	1431 (9.9)	2.8 (2.5, 3.2)	<0.005	5.5 (4.8, 6.4)	<0.005
**Diameter of largest CBD stones on IOC**					
<4 mm (reference)	4116 (28.6)	-	-	-	-
4–8 mm	7939 (55.1)	1.7 (1.5, 2.0)	<0.005	2.3 (1.9, 2.7)	<0.005
>8 mm	2347 (16.3)	4.3 (3.6, 5.0)	<0.005	4.7 (3.9, 5.8)	<0.005
**Common bile duct diameter on IOC**					
<6 mm (reference)	1827 (12.7)	-	-	-	-
6–10 mm	8647 (60)	1.5 (1.2, 1.8)	0.001	1.4 (1.1, 1.8)	0.003
>10 mm	3928 (27.3)	2.9 (2.4, 3.6)	<0.005	2.0 (1.5, 2.5)	<0.005
**Position of CBD stones in common bile duct on IOC**†					
Low CBD stones (reference)	13 212 (91.7)	-	-	-	-
High CBD stones	1190 (8.3)	2.5 (2.1, 2.8)	<0.005	1.9 (1.6, 2.2)	<0.005
**Preoperative symptoms of CBD stones**‡					
No (reference)	6194 (43.0)	-	-	-	-
Yes	8208 (57.0)	1.1 (1.0, 1.3)	0.026	1.0 (0.9, 1.2)	0.839
**Emergency cholecystectomy**					
No (reference)	5990 (41.6)	-	-	-	-
Yes	8412 (58.4)	1.1 (1.0, 1.2)	0.237	1.2 (1.1, 1.4)	0.001
**Gender**					
Female (reference)	9300 (64.6)	-	-	-	-
Male	5102 (35.4)	1.2 (1.1, 1.4)	<0.005	1.1 (1.0, 1.3)	0.042
**Age§**					
Less than cohort median (reference)	6604 (45.9)	-	-	-	
Greater than cohort median	7798 (54.1)	1.5 (1.4, 1.7)	<0.005	1.2 (1.1, 1.4)	0.002

Values in parentheses are percentages unless indicated otherwise;

* values in parentheses are 95 per cent confidence intervals.

† High common bile duct (CBD) stones are CBD stones located above the cystic duct.

‡ Previous or ongoing jaundice or pancreatitis. §Median age for the entire cohort was 51 years. IOC, intraoperative cholangiography.

### Asymptomatic common bile duct stones less than 4 mm in diameter

Of the 2168 patients with small (less than 4 mm) and asymptomatic CBD stones, a total of 121 (5.6 per cent) had a retained CBD stone during the follow-up period (*Fig. S1*). Some 71 of 661 (10.7 per cent) patients that underwent surveillance had a retained stone compared with 50 of 1507 (3.3 per cent) in the intervention group (*Table S6*). The HR for retained stone was 3.3 (95 per cent c.i. 2.3 to 4.7) in the surveillance group and was slightly higher at 3.5 (95 per cent c.i. 2.4 to 5.1) after adjusting for emergency cholecystectomy, common bile duct diameter, stone location, as well as sex and age (*Table 4*).

**Table 4:** Cox proportional hazards analysis with endoscopic retrograde cholangiopancreatography for small retained common bile duct stones

	Patients with asymptomatic CBD stones <4 mm in diameter (*n* = 2168)	**Univariable analysis**		Multivariable analysis	
		Hazard ratio of retained CBD stones*	*P*	Hazard ratio of retained CBD stones*	*P*
**CBD stones management**					
Intervention (reference)	1507 (69.5)	-	-	-	
Surveillance	661 (30.5)	3.3 (2.3, 4.7)	<0.005	3.5 (2.4, 5.1)	<0.005
**Common bile duct diameter on IOC**					
<6 mm	783 (36.1)	-	-	-	
6–10 mm	1262 (58.2)	1.3 (0.9, 1.9)	0.223	1.4 (0.9, 2.1)	0.123
>10 mm	123 (5.7)	2.0 (1.0, 3.9)	0.044	2.3 (1.2, 4.6)	0.019
**Position of CBD stones in common bile duct on IOC**†					
Low CBD stones (reference)	2035 (93.9)	-	-	-	
High CBD stones	133 (6.1)	1.7 (0.9, 3.0)	0.091	1.7 (1.0, 3.2)	0.075
**Emergency cholecystectomy**					
No (reference)	1340 (61.8)	-	-	-	
Yes, emergency cholecystectomy	828 (38.2)	1.2 (0.8, 1.7)	0.299	1.5 (1.0, 2.1)	0.034
**Gender**					
Female (reference)	1603 (73.9)	-	-	-	
Male	565 (26.1)	1.0 (0.7, 1.5)	0.953	0.9 (0.6, 1.3)	0.875
**Age**‡					
Less than cohort median (reference)	1180 (54.4)	-	-	-	
Greater than cohort median	988 (45.6)	1.8 (1.2, 2.5)	0.002	1.6 (1.1, 2.4)	0.011

Values in parentheses are percentages unless indicated otherwise;

* values in parentheses are 95 per cent confidence intervals.

† High common bile duct (CBD) stones are CBD stones located above the cystic duct.

‡ Median age for the entire cohort was 51 years. IOC, intraoperative cholangiography.

## Discussion

This study showed that patients with a CBD stone detected at IOC and left *in situ* had a statistically significant higher risk for retained CBD stones requiring ERCP than that of patients who underwent a planned perioperative intervention for stone removal. A subgroup analysis in patients with small and asymptomatic CBD stones also suggested that these should be removed intraoperatively or shortly after surgery with ERCP. When comparing risk for retained stones between the non-IOC group and the IOC group, the risk was higher in the non-IOC group (HR 1.4, 95 per cent c.i. 1.3 to 1.6).

The study was based on data from the GallRiks registry that has nearly complete (90 per cent) coverage of patients undergoing gallstone surgery or ERCP in Sweden^8^.

The outcome measures only included patients who, for any reason, underwent an unplanned ERCP and had a retained CBD stone. The indication for the ERCP was unknown and the outcome measure could thus include asymptomatic stones discovered during ERCP undertaken for other reasons.

Retained CBD stones of minor clinical relevance and CBD stones that rapidly passed spontaneously were not included, nor were patients with retained stones not undergoing ERCP for other reasons such as CBD stone-related death, too frail to undergo ERCP, and those managed conservatively. This could lead to underestimation of the risk for retained stones. Further studies where these data are cross-referenced with the national patient registry could shed light on this potential bias.

Another weakness of the definition of retained CBD stones was the inclusion of patients with a bile duct stent left *in situ* and who were planned for ERCP later than 7 days after surgery. During ERCP and removal of the stent, minor retained stones, otherwise asymptomatic and never identified if the ERCP had not been undertaken, would have been registered as a terminal event. This is a potential source of bias since delayed ERCP in this case was part of the primary strategy and not due to clinical relevance of the stones. Unfortunately, it was not possible to identify these patients from the registry data. Since this group was included as ‘intervention’ and registered with the terminal event, it could make the intervention appear less favourable.

Although IOC is an accurate diagnostic method for identification of CBD stones^12^, false-negative or -positive test results may occur. Also, the estimation of CBD stone diameter may be inaccurate. However, it is believed that the data from this study are externally valid as the study represents current clinical practice across a nation. Finally, there is a potential selection bias towards the group where no IOC was performed, but there are no data available on the reason why IOC was omitted, other than when it was attempted and unsuccessful.

The decision to choose intervention or surveillance of CBD stone was made by the surgeon and probably depended on previous symptoms and stone size. This could also have caused selection bias. However, the subgroup analysis of small and asymptomatic stones still showed that, regardless of selection and assuming a favourable course, CBD stones believed to be harmless if left in place benefit from removal, thus avoiding the risk for retained stones becoming symptomatic.

Since interventions range from simply flushing or manipulating the CBD stone through the papilla Vateri to open choledochotomy with extraction of the stone, the intervention group was heterogeneous. In the present study, focus on stones left to surveillance compared with any intervention was considered relevant because surveillance is a fundamentally different form of management. Furthermore, surveillance was the preferred strategy in 30.5 per cent (661 of 2168) of the smallest asymptomatic stones.

Findings of the present study are in line with a previous study based on GallRiks data where risk for unfavourable 30-day outcome was greater if CBD stones were left *in situ* without intervention^13^. The British Society of Gastroenterology guidelines also recommend that all CBD stones be removed when identified at cholecystectomy^14^.

However, recently published data suggest a conservative approach for asymptomatic CBD stones, partly because of the risk for complications of ERCP itself^15^. In that study, however, the safer strategy of rendezvous ERCP^10^ was not employed and CBD stone size was not reported.

The results are applicable in the international setting where IOC may not always be a routine examination during cholecystectomy. In many countries IOC is undertaken selectively such as when the anatomy is unclear or when there is strong suspicion of CBD stones^14^. The findings of this study suggest that a substantial proportion of CBD stones that are asymptomatic would remain undetected if IOC were omitted, and that many patients would require subsequent ERCP for extraction of retained CBD stones. In other words, ERCP for retained CBD stones could be avoided if IOC is performed routinely and any stones detected removed intraoperatively or removed shortly after primary surgery using rendezvous ERCP.

The risk of leaving a CBD stone *in situ* must always be weighed against the risk of the intervention performed to remove the stones. It was not within the scope of this study to compare risk for complications, other than retained stones, between intervention and surveillance. What this study does show, however, is that the risk of having to undergo late postoperative ERCP because of retained stone is higher if IOC is omitted or if CBD stones are not managed immediately. Further studies are needed to compare the overall complication risk between surveillance and various methods for clearing CBD stones.

## Funding

Centre for Clinical Research, Uppsala University, Falun, Sweden. The study was supported by a grant from Ruth and Rickard Juhlin Research Foundation. All authors have completed the ICMJE uniform disclosure form. Reporting checklist: the authors have completed the STROBE reporting checklist. Ethics statement: the authors are accountable for all aspects of the work in ensuring that questions related to the accuracy or integrity of any part of the work are appropriately investigated and resolved. The study was conducted in accordance with the Declaration of Helsinki (2013 revision) and informed consent was obtained from all the patients prior to registration in the GallRiks. The study was approved by the Swedish Ethics Review Authority dnr 2019-04224.

## Acknowledgements

The authors thank Peter Cox, Norrköping, Sweden, for skilful linguistic revision. The study was, after careful consideration, not preregistered since it utilizes only previously collected registry data. 

*Disclosure:* The authors declare no conflict of interest.

## Supplementary material

Supplementary material is available at *BJS* online.

## Supplementary Material

znab324_Supplementary_Data
