# Supplementary material for: Intervention versus surveillance in patients with common bile duct stones detected by intraoperative cholangiography: a population-based registry study
Source: Br J Surg. 2021 Oct 13;108(12):1506–12. doi: 10.1093/bjs/znab324 (PMC10364905; doi:10.1093/bjs/znab324)
Supplement: znab324_Supplementary_Data [file znab324_supplementary_data.zip › znab324-suppl_data/Supplementary information.docx]

Supplementary Table 1. All possible outcomes registered under the IOC data field in GallRiks for all patients in the cohort n=132 087.

|  | | Number of patients | Percent of patients | Cumulative Percent |
| --- | --- | --- | --- | --- |
|  | Not performed | 10778 | 8.2 | 8.2 |
|  | Normal finding | 95738 | 72.5 | 80.6 |
|  | Stone | 15351 | 11.6 | 92.3 |
|  | Stenosis/tumor | 470 | .4 | 92.6 |
|  | Other | 2977 | 2.3 | 94.9 |
|  | Incomplete contrast filling | 1473 | 1.1 | 96.0 |
|  | Attempted but unsucessful | 5300 | 4.0 | 100.0 |
|  | Total | 132087 | 100.0 |  |

Supplementary Table 2. Patients with a CBD stone detected by IOC. Patient characteristics

n=14402

|  | | |  | |  |  |
| --- | --- | --- | --- | --- | --- | --- |
|  |  |  | intervention  (n= 12971) | Surveillance  (n=1431) | All patients  (n=14402) |  |
| Age, Median (range) |  | | 54 (78) | 54 (73) | 54 (78) |  |
| Gender n (%) | Male |  | 4633 (35.7)) | 469 (32.8) | 5102 (35.4) |  |
|  | Female |  | 8338 (64.3) | 962 (67.2) | 9300 (64.6) |  |
| Diameter of largest CBDS on IOC  n (%) | >8 mm |  | 2276 (17.5) | 71 (5.0) | 2347 (16.3) |  |
|  | 4 - 8 mm |  | 7482 (57.7) | 457 (31.9) | 7939 (55.1) |  |
|  | <4 mm |  | 3213 (24.8) | 903 (63.1) | 4116 (28.6) |  |
| Common bile duct diameter on IOC  n (%) | >10 mm |  | 3764 (29.0) | 164 (11.5) | 3928 (27.3) |  |
|  | 6 – 10 mm |  | 7819 (60.3) | 828 (57.9) | 8647 (60.0) |  |
|  | <6 mm |  | 1388 (10.7) | 439 (30.7) | 1827 (12.7) |  |
| Highest CBDS  n (%) | low CBDS |  | 11902 (91.8) | 1310 (91.5) | 13212 (91.7) |  |
|  | High CBDS |  | 1069 (8.2) | 121 (8.5) | 1190 (8*.3*) |  |
| Preoperative symptoms of CBDS  n (%) | no |  | 5203 (40.1) | 991 (69.3) | 6194 (43.0) |  |
|  | Yes |  | 7768 (59.9) | 440 (30.7) | 8208 (57) |  |
| Emergency procedure  n (%) | no |  | 5132 (39.6) | 858 (60.0) | 5990 (41.6) |  |
|  | Yes |  | 7839 (60.4) | 573 (40.0) | 8412 (58.4) |  |

Supplementary Table 3. Asymptomatic patients with CBD stones less than 4 mm in diameter. Patient characteristics. N=2168

|  | |  | |  |  |
| --- | --- | --- | --- | --- | --- |
|  |  | Intervention  n=1507 | Surveillance  n=661 | All patients  n=2168 |  |
| Age | Median (Range) | 49 (77) | 50 (70) | 49 (77) |  |
| Gender n (%) | Male | 383 (25.4) | 182 (27.5) | 565 (26.1) |  |
|  | Female | 1124 (74.6) | 479 (72.5) | 1603 (73.9) |  |
| Diameter of largest CBD Stones on IOC n (%) | <4 mm | 1507 (100) | 661 (100) | 2168 (100) |  |
| Common bile duct diameter on IOC  n (%) | >10 mm | 101 (6.7) | 22 (3.3) | 123 (5.7) |  |
|  | 6 -10 mm | 911 (60.5) | 351 (53.1) | 1262 (58.2) |  |
|  | <6 mm | 495 (32.8) | 288 (43.6) | 783 (36.1) |  |
| Highest CBD Stones  n (%) | low CBDS | 1430 (94.9) | 605 (91.5) | 2035 (93.9) |  |
|  | high CBDS | 77 (5.1) | 56 (8.5) | 133 (6.1) |  |
| Preoperative symptoms of CBD Stones  n (%) | no | 1507 (100) | 661 (100) | 2168 (100) |  |
| Emergency cholecystectomy  n (%) | no | 872 (57.9) | 468 (70.8) | 1340 (61.8) |  |
|  | yes | 635 (42.1) | 193 (29.2) | 828 (38.2) |  |

Supplementary Table 4. Number of patients with retained CBD stone per management group for patients with IOC compared to IOC not performed. All patients in the cohort n=132 087

|  | |  | | All patients |
| --- | --- | --- | --- | --- |
|  |  | IOC  performed | IOC not performed |  |
| Retained CBD stone | No | 113718 | 15678 | 129396 |
|  | Yes | 2291 | 400 | 2691 |
| Total | | 116009 | 16078 | 132087 |

Supplementary Table 5. Number of patients with retained CBD stone per management group for all patients with CBD stones identified by IOC. n=14 402.

|  | | | | |
| --- | --- | --- | --- | --- |
|  | |  | | All patients |
|  |  | Intervention | Surverillance |  |
| Retained CBD stone | No | 11944 | 1138 | 13082 |
|  | Yes | 1027 | 293 | 1320 |
| Total | | 12971 | 1431 | 14402 |

Supplementary Table 6. Number of patients with retained CBD stone per management group for asymptomatic patients with CBD stones less than 4 mm in diameter. n=2168.

|  | | | | |
| --- | --- | --- | --- | --- |
|  | | | | |
|  | |  | | Total |
|  |  | Intervention | Surveillance |  |
| Retained CBD stone | No | 1457 | 590 | 2047 |
|  | Yes | 50 | 71 | 121 |
| Total | | 1507 | 661 | 2168 |
